# Learning Geometric Feature Embedding with Transformers for Image Matching

**DOI:** 10.3390/s22249882

**Published:** 2022-12-15

**Authors:** Xiaohu Nan, Lei Ding

**Affiliations:** 1 Shanghai Institute of Technical Physics, Chinese Academy of Sciences, Shanghai 200083, China; 2 University of Chinese Academy of Sciences, Beijing 100049, China; 3 Key Laboratory of Infrared System Detection and Imaging Technology, Chinese Academy of Sciences, Shanghai 200083, China

**Keywords:** local feature matching, attention, deep learning

## Abstract

Local feature matching is a part of many large vision tasks. Local feature matching usually consists of three parts: feature detection, description, and matching. The matching task usually serves a downstream task, such as camera pose estimation, so geometric information is crucial for the matching task. We propose the geometric feature embedding matching method (GFM) for local feature matching. We propose the adaptive keypoint geometric embedding module dynamic adjust keypoint position information and the orientation geometric embedding displayed modeling of geometric information about rotation. Subsequently, we interleave the use of self-attention and cross-attention for local feature enhancement. The predicted correspondences are multiplied by the local features. The correspondences are solved by computing dual-softmax. An intuitive human extraction and matching scheme is implemented. In order to verify the effectiveness of our proposed method, we performed validation on three datasets (MegaDepth, Hpatches, Aachen Day-Night v1.1) according to their respective metrics, and the results showed that our method achieved satisfactory results in all scenes.

## 1. Introduction

In 3D computer vision tasks such as simultaneous localization and mapping (SLAM) [1], structure from motion (SfM) [2,3] and visual localization [4,5,6], the correspondence between pixels of an image is important for estimating 3D structures and camera poses. This correspondence is usually estimated by matching local features, a process known as local feature matching, which allows the recovery of high-dimensional structures from low-dimensional image pairs. Large viewpoint position variations, angle changes, occlusions, and blurring are the main factors that limit the local feature matching performance.

The most widely adopted local feature matching pipeline consists of three consecutive steps: feature detection, feature description, and feature matching. For the two images to be matched, in the detection phase, significant points are first detected from each image as interest points. Then local descriptors are extracted in the neighboring regions of these interest points. The feature detection and description phases produce interest points with descriptors, and the point-to-point correspondence is eventually found by a nearest neighbor search or more sophisticated matching algorithms. However, calculating matching relationships using a nearest neighbor search is prone to errors due to the ambiguity of local descriptors. Especially in the presence of large variations in viewpoints, methods such as hand-designed outlier filtering can mitigate this problem to some extent.

Some recent research methods try to solve this problem by dense matching. The matches with high confidence scores are selected from the dense matches to obtain more reliable matches. Some other approaches solve the matching problem using a larger image context. For example, SuperGlue [7] introduces graph neural networks [8] for matching between local features on images. The edges of the graph connect keypoints at all locations, allowing for extensive contextual inference. The graph convolutional network has greater perceptual field information than convolutional neural networks. Moreover, it can capture information with long range, thus having a wider range of global information.

However, some methods of SuperGlue et al. [7,9]. do not take into account geometry-related information, which can be very helpful to viewpoint changes. We rethink the problem of geometric embedding information in two ways: (i) In a pair of images, the positions of keypoints describing the same localities can be offset or not correspond to each other, leading to the existence of unmatched keypoints in the pair. (ii) For pairs of images with large changes in camera pose, many false matches are observed.

Based on the above observation, for the above thought (i), we believe that accurate keypoint locations need to learn offsets that focus on keypoint regions. And from this idea, we propose a simple and effective method, the keypoint adaptation module. We use a simple convolutional neural network to learn pixel-level spatial transformations as an extension of the keypoint location. It further facilitates the information exchange after the change of viewpoint position. For the above reflection (ii), we propose a simple and effective method, the local feature affine module. We use convolutional neural networks to learn the spatial transform of local features, find the main direction of local features from the spatial transform, and then encode them using a sine-cosine encoder. The properties of the geometric affine transform are assigned to the local features.

**Figure 1:**
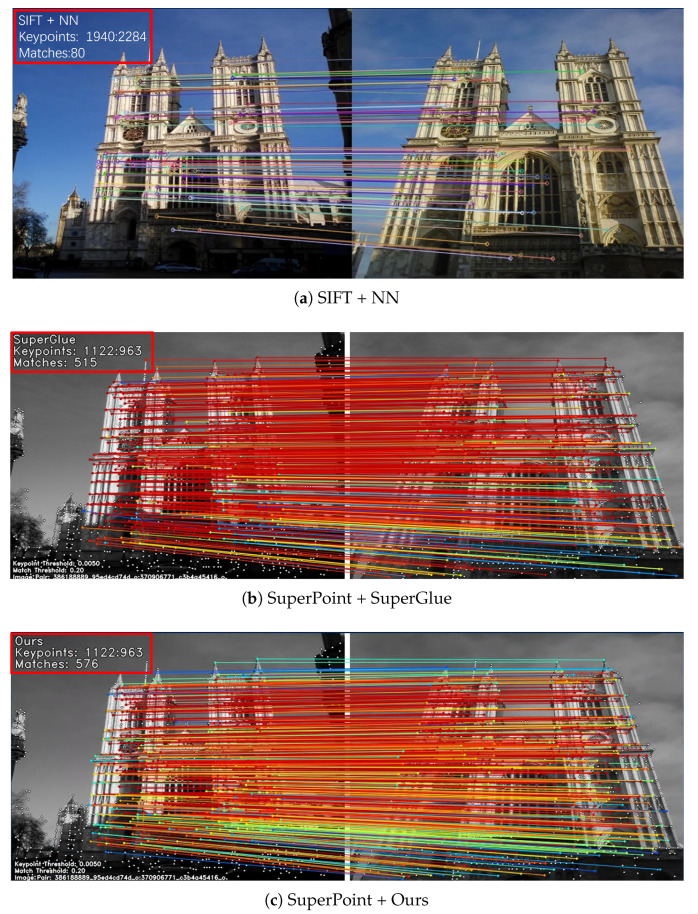
Visualization of different methods. For the same image pairs, we use three different methods to visualize the effect. subfigure (**a**) represents SIFT + NN, SIFT is a hand-craft feature, subfigure (**b**) represents SuperPoint + SuperGlue, SuperGlue is a learning-based matcher, and subfigure (**c**) is SuperPoint + GFM, which is our proposed method. The number of keypoints and the number of matching pairs are marked in the upper left corner of all images using red rectangular boxes. Comparing subfigure (**a**) and subfigure (**c**), we can see that our method can obtain more dense and robust matching pairs compared to traditional matching. Comparing subfigure (**b**) and subfigure (**c**), we can see that our method is more advantageous in obtaining the number of correct matching pairs.

Combining the above reasons, we propose Geometric Feature Embedding Matching (GFM), inspired by SuperGlue. We use Transformer with self-attention and cross-attention layers to handle dense local features. Given a pair of local features, containing keypoints, descriptors, and local feature confidence, the GFM outputs the correspondence between local features. The visualized effect is shown in Figure 1.

We perform the local feature matching estimation and camera pose estimation tasks on three publicly available datasets (MegaDepth [10], Hpatches [11], Aachen Day-Night v1.1 [12]) and evaluate the proposed method. The experimental results show that the feature matching performance of our method outperforms other methods. The subsequent chapters are organized as follows: Section 2 compares the related work in the field of local feature matching. Section 3 focuses on our method. Section 4 focuses on the experimental results and the corresponding analysis. Section 5 describes the results of the ablation experiments and analyzes the effectiveness of the proposed method, and Section 6 concludes the whole paper.

The main contributions of our approach to the local feature matching task are as follows: (i). For the case of viewpoint position offset, we propose the adaptive keypoint geometric embedding module that learns the 2D offsets of keypoints by a simple convolutional neural network as an extension of keypoints to give a more accurate representation of keypoints for information flow. (ii). For the case of large angular transformations of viewpoints, we propose the local feature affine module that uses the 2D offsets learned by local features to obtain the affine transformation encoding, giving the geometric information of the affine transformation to the viewpoints. (iii) Our proposed GFM method achieves satisfactory results on three publicly available datasets, including MegaDepth, Hpatches, and Aachen Day-Night v1.1.

## 2. Related Work

Conventional Feature Matching. These are the main methods for local feature matching. Many well-known traditional hand-craft methods have achieved good performance on local features, and hand-craft features such as SIFT [13] and ORB [14] are still widely used in many tasks today. In contrast to traditional hand-craft features, learning-based methods perform better with changes in viewpoint and local illumination, etc. Recent learning-based methods focus on learning local features using convolutional neural networks (CNNs). MagicPoint [15] is a well-known learning-based local feature that uses a hand-crafted detector-based design to improve performance in all aspects. SuperPoint [16] proposes self-supervised learning using a single-response matrix based on MagicPoint. D2Net [17] obtains valid keypoints by detecting local maxima of CNN features. R2D2 [18] adapts extended convolution to maintain image resolution and predict each keypoint and descriptor. Since the nearest neighbor search is prone to outliers, some methods [19,20] study the neighborhood consistency and others learn to filter outliers.

Learning-based Feature Matching. NCNet [21] proposes to learn the correspondence directly in an end-to-end manner. It uses all points in the feature map to compute the 4D tensor to construct all possible matches, and uses 4D convolution to normalize the 4D tensor. Sparse-NCNet [22] notices that the computation of 4D convolution brings a great challenge to memory, so it solves this problem by introducing sparse convolution, which eventually makes the computation more efficient. The main idea of DRC-Net [9] remains unchanged. DRC-Net obtains two CNN feature maps with different resolutions, generates two 4D matching tensors, and fuses them to achieve high-confidence feature matching. All the above methods obtain two CNN feature maps with different resolutions, generate two 4D matching tensors, and fuse them to achieve high confidence feature matching and filter by confidence. Recently the combination of geometric information and Transformer or GCN will also be utilized in point cloud registration and embedding [23,24,25], and these methods are some inspiring ideas for image matching.

However, due to the limitation of the acceptance domain of the convolutional neural network, it lacks the global contextual connection to distinguish weak textures or locally similar regions. For this reason, SuperGlue [7] replaces the nearest neighbor search approach and represents a sparse matching network which uses descriptors to manipulate keypoints. Using a graph convolutional neural network, all local features can interact with each other so that an exact match can be obtained. COTR [26] directly manipulates images in a coarse to fine manner. It is a Transformer network that predicts the correspondence of the query keypoints in one image in the second image. It can be seen that it considers the global information. Some of the subsequent methods [27] also continue the idea of using the Transformer to learn the overall correspondence. While the conventional feature matching method divides local feature matching into different phases, the learning-based feature matching method integrates the three phases into a whole. Our proposed method requires already extracted features as input and focuses on learning how to get matching relationships, so our proposed method belongs to conventional feature matching.

## 3. Method

**Figure 2:**
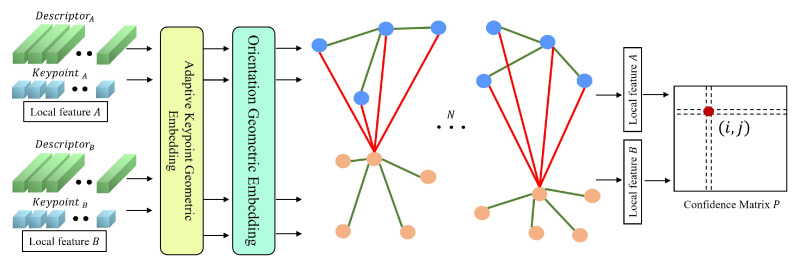
Overview of the proposed method. Our proposed method has four main steps. 1. The offset map is calculated by the local features. Keypoints are adaptively adjusted according to the offset map. 2. The main orientation is obtained with the offset map, and encoded as geometric information with sine-cosine encode values. The responses of local features and geometric information are embedded in high-dimensional features. Then, they are fused with descriptors. 3. The descriptors are processed by the Transformer module and the descriptors with contextual information are obtained. The Transformer module has *L* self-attention layers and cross-attention layers. 4. The matching score is calculated by the similarity of descriptors, and then dual-softmax is used to calculate the corresponding relationship between local features.

Given image pairs $I_{A}$ and $I_{B}$, there exists a set of keypoint locations *p* and visual descriptors *d* for each image, where the keypoint locations are composed of *x*, *y* coordinate point locations $p_{i}={\left(x,y\right)}_{i}$. The visual descriptors $d_{i}\in R^{D}$. The input to our method is the keypoint locations and visual descriptors, and the output is the correspondence of a pair of local features. Figure 2 illustrates the entire flow of the method. A pair of already extracted local features (Following SuperGlue [7], we also use the SuperPoint [16] network for feature extraction and feature description, but the source of the input local feature is flexible. For example, hand-craft descriptors can also be used). The location of the keypoints is extended by the keypoint adaptation module, and the local feature affine module calculates the principal direction of the keypoints, which is embedded in the affine transformation matrix by the sine-cosine function to give the information of the keypoint geometry. The Transformer module uses the attention mechanism to propagate the local feature information and finally computes the matching confidence matrix.

### 3.1. Adaptive Keypoint Geometric Embedding

**Figure 3:**
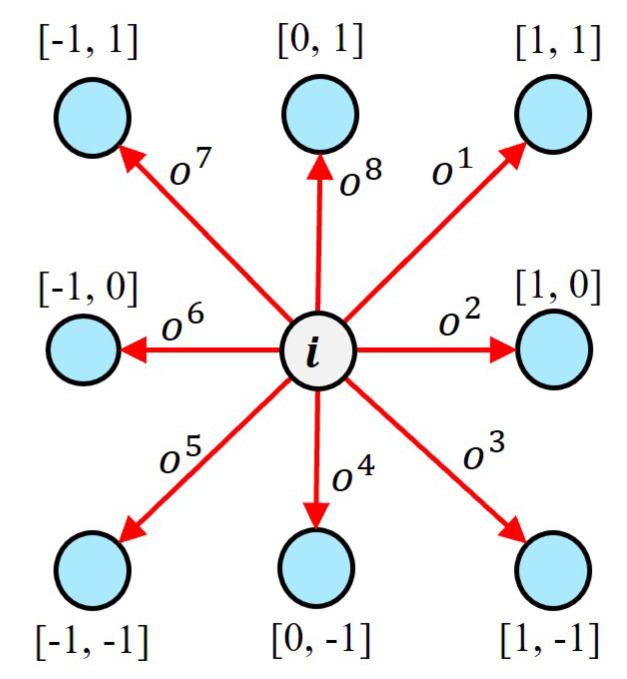
Adaptive adjustment of the keypoint. For local features, we use a simple convolutional network to predict the offset map, which contains offsets on eight orientations. Different prediction weights are weighted to the offset maps separately, and the keypoint is dynamically adjusted by the weighted offset values. The keypoint location information is refined at the pixel-level.

Different feature extraction methods apply to different scenarios, and it is difficult to have a universal feature extractor, so the keypoint positions may not be very accurate or applicable to this stage of matching. Our idea is that we can learn the transformations about the geometry from the high-dimensional descriptors, which can be used to adjust the keypoint positions. The method uses the predicted transformation matrix and the standard position of the keypoints to calculate the position offset of the keypoints as well as the surrounding keypoints, and subsequently adjusts the position of the keypoints and the surrounding keypoints according to the offset. We refine the keypoint locations at the pixel level by learning spatial transformations. The architecture of the module is illustrated in Figure 3. We use a simple convolutional network to capture the spatial relationships of features by predicting *K* transformation matrices from *K* descriptors, estimating the transformations present at each keypoint. The spatial transformations contain all the keypoint offset mappings *O*, which are obtained by a simple convolutional network calculation: $O_{i}=F_{i}\left(d_{i}\right)$, where $F_{i}$ is the *i*-th predicted offset mapping, $d_{i}$ represents *i*-th local descriptor.

Since the original keypoint region can have inaccuracies that prevent the descriptor from being highly focused on the keypoints, we add these pixel-level offsets to the keypoint locations to obtain a more accurate keypoint location. The formula is shown in Equation (1).

(1) $$p_{i}^{adp}=p_{i}+W_{i}\cdot o_{s}^{i},$$

where $p_{i}^{adp}$ represents the *i*-th adjusted keypoint position, $p_{i}$ represents the origin keypoint position, $W_{i}$ is the weight of the convolution kernel, $o_{s}^{i}$ represents all offsets of the *i*-th keypoint.

Specifically, the offsets {$o_{s1}^{p}$, $o_{s2}^{p}$, ..., $o_{s8}^{p}$} (represented by the 2 × 8 matrix $O_{s}^{p}$) extend the spatial transformation network from a global to a pixel-wise approach in a parametric way. For each descriptor, we estimate a spatial transformation matrix $A^{p}\in R^{2\times 2}$ and a translation vector $t\in R^{2\times 1}$, and then compute the offset map for $p_{r}$ (standard 2 × 8 location coordinates), as shown in Equation (2).

(2) $$O_{s}^{p}=A^{p}\cdot p_{r}+t,$$

specifically, in formal terms, $p_{r}$ is described as follows:

(3) $$p_{r}=\left[\begin{array}{cccccccc} -1 & 0 & 1 & -1 & 1 & -1 & 0 & 1\\ -1 & -1 & -1 & 0 & 0 & 1 & 1 & 1 \end{array}\right]\;.$$

$p_{r}$ represents the position of eight neighboring points around a keypoint.

### 3.2. Orientation Geometric Embedding

**Figure 4:**
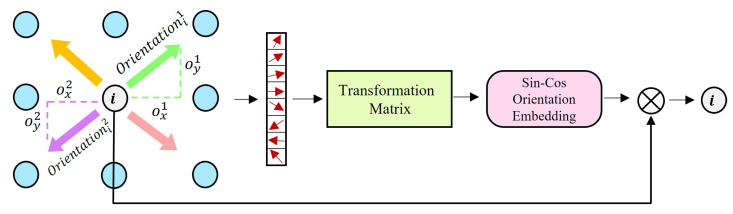
Geometric Orientation Embedding. The eight orientations around the keypoint are calculated from the offset map, and the maximum value is taken as the main orientations of the local feature. Then the transformation matrix is obtained by the principal orientation transformation. The transformation matrix is encoded as sine-cosine information, which is embedded in the high-dimensional features along with the response values.

We consider that keypoints maintain robustness to such cases as rotations in the process of establishing correspondence, so we propose a simple module for geometric orientations embedding of keypoint that explicitly models the orientation of keypoints and captures the relationship between the orientation of keypoints and space. The keypoints are motivated to learn spontaneously the geometric estimation properties with respect to rotations. We calculate the eight orientations around a keypoint based on the offset map. In the form shown in Equation (4). We convert the orientation into information related to geometric transformations called transformation matrix in the shape of 2 × 2. Formally, it is given by Equation (5). In order for the geometric information to propagate in the correct way, it needs to satisfy the properties of the transformation matrix, the formula is expressed as A·D−C·D=1. We encode the transformation matrix with geometric information into sine-cosine information, which is formally shown in Equations (6) and (7). Subsequently, we concatenate the orientation encoding information and the response of the keypoints called geometric-response information ψ, in the form shown in Equation (8). We use a multilayer perceptron (MLP) to generate a positional encoding for all keypoints in the image and the corresponding geometric-response information ψ, and embed it in a high-dimensional vector. This encoding allows us to combine geometric information, response information and visual information and propagate them for joint training. The multilayer perceptron is composed of three simple modules including a fully connected layer, a batch normalization layer, and a Rectified Linear Unit (ReLU) function. For the initialization of the transformation matrix, we define it as an identity matrix. The architecture of the module is illustrated in Figure 4.

(4) $$\theta_{i}=\frac{O_{y}^{i}}{O_{x}^{i}}.$$

where $O_{y}^{i}$ represents the offset of the y-axis, $O_{x}^{i}$ represents the offset of the x-axis.

(5) $$M_{transform}=\left[\begin{array}{cc} A & B\\ C & D \end{array}\right]\;,$$

where A,B,C,D are the values that the network learns and updates during training.

(6) $$\varphi_{sin}=A/\sqrt{A\cdot D-C\cdot D},$$

where $\varphi_{sin}$ represents the sine encoding of the keypoint orientation.

(7) $$\varphi_{cos}=B/\sqrt{A\cdot D-C\cdot D},$$

where $\varphi_{cos}$ represents the cosine encoding of the keypoint orientation.

(8) $$\psi =concat(r,\varphi_{sin},\varphi_{cos}),$$

where *r* respresents the response of keypoints.

### 3.3. Transformer

**Figure 5:**
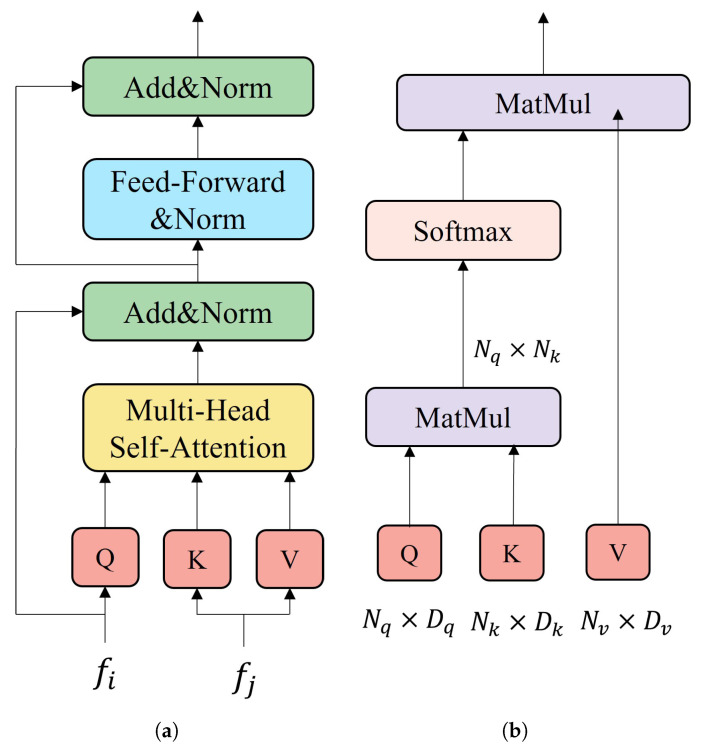
Transformer encoder and dot-product attention layer. The Transformer encoder is made up of *L* identical layers stacked on top of each other. Each layer has two sub-layers. The first layer is a multi-head self-attention mechanism and the second layer is a simple, fully connected feedforward network. A residual connection is used on both sublayers and followed by layer normalization. The input of the dot-product attention includes the query, key, and value. We compute the dot product of the query with the key and apply a softmax function to get the weights of the value. (**a**) Transformer encoder. (**b**) Dot-product attention layer.

The embedding of a high-dimensional local feature $f_{i}$ is computed from its keypoint coordinates $x_{i}$, confidence $c_{i}$ and descriptor $d_{i}$. The formal representation is shown in Equation (9). We use the Transformer structure for message propagation at all keypoints. The Transformer encoder consists of sequentially connected encoder layers, and Figure 5a shows the structure of the encoder layers. We also use self- and cross-attention mechanism to focus attention on specific locations, with self-attention focusing on keypoints within the same image. Cross-attention focuses on keypoints from different images. This allows the Transformer to consider spatial location, determinism and visual appearance during the matching process.

(9) $$f_{i}=d_{i}+F_{encode}\left(\left[x_{i}∥c_{i}\right]\right),$$

where ∥ denotes concatenation and $F_{encode}$ is a multilayer perceptron (MLP). This encodes the keypoints and their confidence into the high-dimensional space of descriptors. This positional encoding facilitates spatial learning [28].

The Transformer encoder is responsible for the propagation of the messages, which is actually the interaction between keypoints. The purpose is to make the matching keypoints closer in the description subspace and the unrelated keypoints keep a larger distance. We set up the Transformer with a total of *L* layers, each layer *l* corresponding to the exchange of messages between keypoints. The layers alternate between layers using self-attention and cross-attention for messaging. Equation (10) formally expresses how the local features are updated during the iterative process.

(10) $$f_{i}^{\left(l+1\right)}=f_{i}^{l}+F_{update}^{l}\left(\left[f_{i}^{l}∥m_{\varepsilon \overset{}{\to }i}^{l}\right]\right),$$

where $m_{\varepsilon \overset{}{\to }i}^{l}$ is the result of aggregating keypoint *i* and all keypoints information by self- or cross-attention. $F_{update}$ is a multilayer perceptron.

The attention mechanism, which combines all messages for keypoints *i* into a single message $m_{\varepsilon \overset{}{\to }i}^{l}$, is computed utilizing dot-product attention in Transfomer. The input vector of the dot-product attention is defined as the query *Q*, the key *K* and the value *V*, where the query *Q* is a high-dimensional representation of the linear projection of the keypoint *i*, and the key *K* and the value *V* are high-dimensional representations of the message source (i.e. keypoint *j*). The key *K* and value *V* in the self-attention information are likewise high-dimensional representations of the linear projection of keypoint *i*, while the key *K* and value *V* in the cross-attention information are obtained by the linear projection of keypoints of all other images. Formally, it is described by Equation (12). The dot-product of query *Q* and key *K* calculates the attention weights, and value *V* retrieves valid information based on the attention weights. The dot-product attention mechanism is shown in Figure 5b. Formally it is described by Equation (13).

It is important to note that the softmax-based weighting algorithm is robust to changes in the number of input views as well as changes in the number of keypoints in layers updated along cross-attention. After the completion of the message passing iteration, the linear projection aims to obtain the global context descriptors for all keypoints. The mathematical description is shown in Equation (11).

(11) $$f_{i}=W_{linear}^{l}f_{i}+b_{4},$$

where $W_{linear}^{l}$ represents the weight and *b* represents the bias. $f_{i}$ is the iteration descriptor assigned to the next layer.

Intuitively looking at the matching problem, when people find an uncertain keypoint, people will repeatedly look at the contextual features around the keypoint, which is used to assist in determining the positive match. This action is actually an iterative process, and as with most methods using the Transformer, the number of layers of the Transformer encoder *L* is set to 9.

(12) $$\begin{array}{c} q_{i}^{l}=W_{1}^{l}f_{i}+b_{1},\\ k_{i}^{l}=W_{2}^{l}f_{j}+b_{2},\\ v_{i}^{l}=W_{3}^{l}f_{j}+b_{3}, \end{array}$$

(13) $$m_{\varepsilon \overset{}{\to }i}^{l}=softmax\left(Q^{l}\cdot {\left(K^{l}\right)}^{T}\right)V^{l}.$$

### 3.4. Establish Correspondences

The assignment matrix is calculated in SuperGlue using optimal transport, which represents the probability that a keypoint is likely to be matched, and it should be noted that a keypoint can only be matched with at most one keypoint in another image. From the Loftr [27] we know that dual-softmax (DS) is used to compute the assignment matrix with the same effect as optimal transport, and we choose the easier one to implement a dual-softmax operation to compute the assignment matrix. Briefly, the matching confidence matrix is calculated between two high-dimensional context descriptors by $S\left(i,j\right)=d_{A}\cdot d_{B}$. We can apply the softmax operation on the two dimensions of *S*, and can calculate the soft mutual neighbor matching probability. Formally, the matching probability matrix *P* is represented by the Equation (14).

(14) $$P\left(i,j\right)=softmax{\left(S\left(i,\cdot \right)\right)}_{j}\cdot softmax{\left(S\left(\cdot ,j\right)\right)}_{i}.$$

Based on the calculated matching probability matrix *P*, the matches with confidence higher than μ are selected. Due to the existence of the principle that at most one keypoint in another image can be matched between keypoints, we use the mutual nearest neighbor criterion (MNN) to filter the wrong matches and finally obtain the matching relationship. The matching relationship prediction can be expressed formally as:

(15) M={(i,j)|∀(i,j)∈MNN(P),P>μ}.

### 3.5. Supervision

We define the loss function as the negative log-likelihood loss of the matching probability matrix P after the dual-softmax calculation. The loss function is described formally as shown in Equation (16). During the training process we compute the ground-truh corresdence using camera poses and depth maps. Specifically, we define the mutual closest proximity of two sets of keypoints set as the ground-truh corresdence $M_{gt}$, where the closest proximity between two sets of keypoints is obtained by computing the reprojection error using the keypoint locations.

(16) $$L=-\sum_{\left(i,j\right)\in M_{gt}}logP\left(i,j\right),$$

### 3.6. Implementation Details

We train the model on the MegaDepth dataset with an optimizer of Adam, an initial learning rate of $1.0e^{-4}$, a batch size of 16, and 100 epochs on an Nvidia Tesla V100 GPU. The number of layers *L* of the Transformer is chosen to be 9, the matching score threshold μ is chosen to be 0.2, and the image sizes are chosen to be 720 and 960 in height and width, respectively. At runtime, we select 1024 features of one image, and limit the resolution of the image pair to 640×480. We process a pair of images in 85 ms.

## 4. Experiments and Results

To validate the effectiveness of our method, we evaluate it on three open datasets, including MegaDepth [10], Hpatches [11], and Aachen Day-Night v1.1 [12]. We use their respective validation methods. A common strategy for evaluating local feature matching methods is to measure their performance in downstream tasks. On the MegaDepth dataset, we follow the SuperGlue approach and use the relative pose estimation task because MegaDepth can provide true pose information between image pairs. On HPatches dataset, we choose the task of estimating the homography matrix, since the Hpatches dataset provides the true homography matrix. On Aachen Day-Night v1.1, we perform the relative pose estimation task under different thresholds.

### 4.1. Outdoor Relative Pose Estimation

Outdoor image sequences have a class of challenges such as illumination, rotation, and occlusion, so we train and evaluate GFM for pose estimation in outdoor environments.

**Dataset**. The MegaDepth [10] dataset includes about 1 million Internet images, divided into a total of 196 different outdoor scenes. The authors also provide sparse reconstruction from COLMAP [3] and depth maps computed from multi-view stereo. We chose half of the validation scenes from SuperGlue for the evaluation. We use the same evaluation environment for all methods, and we select 18 scenes as the validation dataset, and take the top 50 image pairs for each validation set for evaluation. We adjust the width and height of the images to 720 and 960, respectively.

**Metrics.** We use the AUC of the pose error at the ($5^{\circ }$, $10^{\circ }$, $20^{\circ }$) threshold, the matching accuracy, and the matching score as the reported values. Among them, the maximum values of the translation error and the angular error are noted as the pose error. In order to recover the camera pose, we solve the fundamental matrix from the predicted correspondence using the RANSAC method. On the other hand, we aim to analyze the performance of each method on the MedaDepth validation set for a single keypoint matching. We use matching precision (Precision), formally expressed as Equation (17), and matching score (MS) as metrics, formally expressed as Equation (18), where the corresponding epipolar line error is less than a threshold value of $5e^{-4}$ and is considered as a correct match.

(17) $$Precision=\frac{TruePositives}{PredictedMatches}$$

(18) $$MatchingScore=\frac{TruePositives}{TotalKeypionts}$$

**Result.** We use some methods with higher performance to compare with our proposed methods, including R2D2 [18], DISK [29], DRC-Net [9], SuperPoint [16], and Superglue [7]. We do not add any remaining processing for the above methods. All baseline methods use originally trained weights to calculate the ground truth pose and depth. All baseline methods are based on the nearest neighbor [30] (NN) matcher and the default outlier rejection method. As shown in Table 1, our method outperforms DRC-Net [9] in all metrics, the results prove the effectiveness of the Transformer.

**Figure 6:**
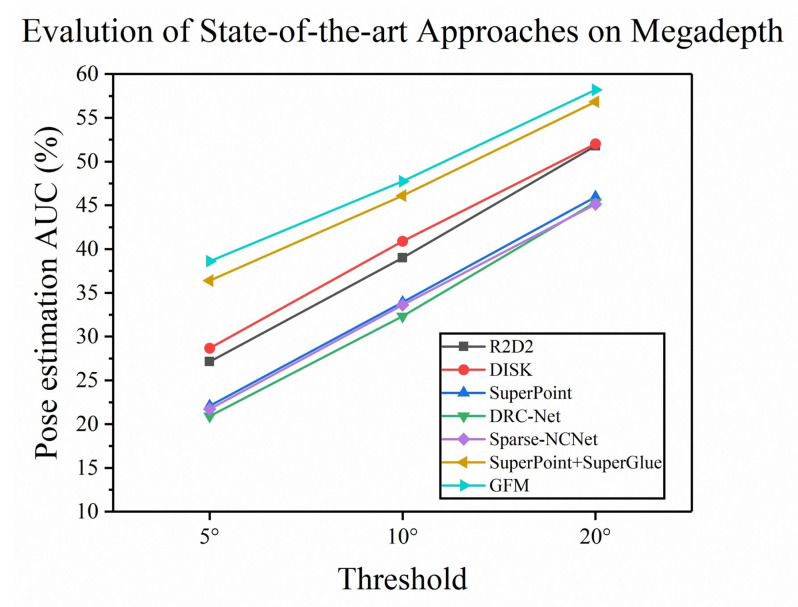
Pose estimation on MegaDepth. Different colors represent different methods, and we show the AUC values for each method at different pose error thresholds ($5^{\circ }$, $10^{\circ }$, $20^{\circ }$).

Compared to a feature-based method such as DISK [29], which uses the reinforcement learning strategy to produce features, DISK produces dense features, but since it does not use a detector with global context and does not take some geometric information into account, the method performs 6.85% lower than our proposed method in terms of AUC@$10^{\circ }$ metric on the MegaDepth dataset. Because we capture the long-range perceptual field with the Transformer mechanism, which has a good effect on the enhancement of local features, and we use descriptors to enhance the geometric properties, which makes our method perform better. Compared with SuperGlue [7], the proposed method exceeds 2.42% in matching accuracy, which proves that the adjustment of keypoint geometric position and the rotational geometric information of keypoints is beneficial to improving the matching accuracy. The performance advantages and disadvantages of each method for solving the poses can be seen qualitatively in Figure 6.

### 4.2. Homography Estimation

We perform a large-scale homography estimation experiment using real images and synthetic homographies with both robust (RANSAC) and non-robust (DLT) estimators.

**Dataset**. HPatches is a widely adopted benchmark for homography estimation on the local feature matching task. HPatches contains 52 sequences evaluating matching performance under significant illumination changes and another 56 sequences exhibit the large view-angle variation cases. For each image sequence, one reference image is included, corresponding to 5 images and the respective homography matrix. Given the ground-truth homography matrix, we inherit the evaluation metrics in computational correctness of homography estimation.

**Metric.** In each test sequence, a reference image is matched with the remaining 5 images. The width and height of all images are adjusted to 480 and 640. For a pair of images, we estimate the homography matrix using the OpenCV and RANSAC [31] method after calculating the correspondence. The estimated homography matrix and the ground-truth homography matrix are then used to calculate the angular error between the images, and the AUC of the error accumulation curve at the (3 pixels, 5 pixels, 10 pixels) threshold is reported.

**Figure 7:**
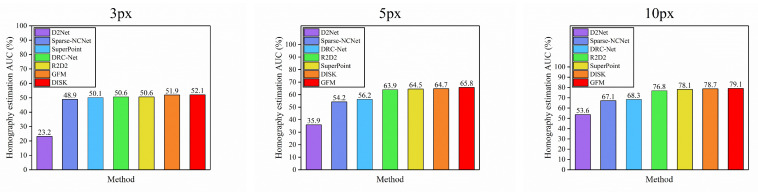
Homography estimation on Hpatches. The three graphs represent the AUC values of each method with a threshold of 3 px, 5 px, and 10 px. Different colored rectangles represent different baseline methods.

**Results.** Our method is compared with other baselines under the same conditions. For local features, we extract up to 2k keypoints for detector-based local feature methods, including D2Net [17], R2D2 [18], etc. For the detector-free matcher, we choose DRC-Net [9] and Sparse-NCNet [22]. It can be seen in Table 2 that our method outperforms other baselines for all pose error thresholds, specifically, the SuperPoint [16] is a self-supervised feature extractor that utilizes an adaptive homography matrix to learn some simple geometric transformations during feature production. Our method learns some complex geometric property variations in features. So our method exceeds 1.3% at 5 px. Compared to Sparse-NCNet [22], our method exceeds 11.6% at 5 px. The reason for this is that Sparse-NCNet is an end-to-end method, but it considers only local features in building the 4D spatial tensor, and these local features are not well adapted to complex scenes, such as scenes with varying illumination or large homophobic changes. Our method takes into account the global context, so it can capture features over long-range distances, and adds geometric property variations, which can be well adapted to scenes with homophobic changes. For DRC-Net [9], our method exceeds 10% at 10 px threshold, which shows that accurate local feature information is important for the local feature matching task. Figure 7 qualitatively describes the accumulative error curve of pose under the threshold of three pixels. Our method performs well on all three pixel thresholds.

### 4.3. Aachen Day-Night v1.1 Dataset

**Dataset.** The Aachen Day-Night dataset, based on the original Aachen dataset, depicts the old city of Aachen, Germany. The scene representations of the database images used to construct the reference are all taken in the daytime. This dataset provides query images taken during the day and night. All the query pictures are taken by the mobile phone camera, that is, the Aachen Day-Night dataset takes into account the use of mobile devices for localization of the scene. The dataset also provides additional daytime image representations that are not part of the reference scene. Although no ground truth camera poses are provided for these images, they can be used as benchmarks.

**Metrics.** We used the cumulative AUC of pose error at (0.25 m, $2^{\circ }$)/(0.5 m, $5^{\circ }$)/(5 m, $10^{\circ }$) thresholds to make a report. We calculated the correspondence between 2D points (the 2D points with the correct correspondence are called valid keypoints), and then solved the pose error by using the correspondence between 2D valid keypoints and 3D points, as well as the rejection of the outliers by the RANSAC method.

**Results.** It can be seen from Table 3 that our method performs better than the other baseline methods in the daytime with illumination. It can be demonstrated that the additional information of geometry is a crucial factor in enhancing the descriptors, and the performance is better than the other baseline methods in all cases except at night when the performance is slightly weaker than that of the Loftr [27] in the 0.25 m limit.

**Figure 8:**
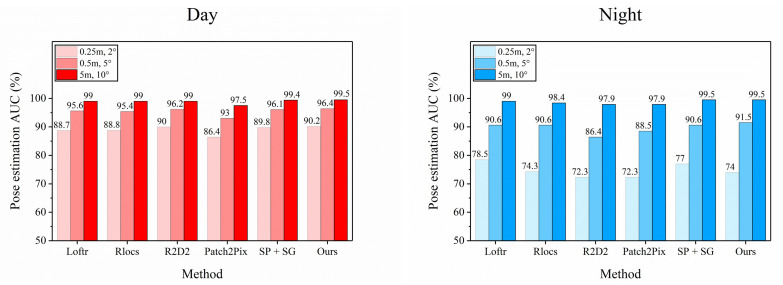
Visual Localization on Aachen Day-Night v1.1. The above two graphs show the performance of each baseline method in the data set under both day and night constraints. The color is divided into three levels according to the transparency. The lightest rectangle corresponds to the pose estimation threshold of (0.25 m, $2^{\circ }$), and the darkest rectangle corresponds to the pose estimation threshold of (5 m, $10^{\circ }$). The middle rectangle corresponds to (0.5 m, $5^{\circ }$).

R2D2 [18] is a method that combines feature repeatability and feature reliability, combining feature detection and extraction, but this method is not able to make good judgments for some environments with large changes in viewpoint. Our method can make some adjustments to the features using the feature adaptivity module and adding some geometric information, so our method surpasses R2D2 in all conditions. These results can prove that our method performs well under both day and night lighting conditions. Figure 8 qualitatively illustrates all baseline methods in both day and night conditions

## 5. Ablation Experiments

To verify the effectiveness of our proposed modules, we conduct experiments on the MegaDepth dataset for each of the two modules, including the adaptive keypoint geometric embedding (AKGE) and orientation geometric Embedding (OGE), but this one is mainly focused on a different variant of the overall approach. The results of ablation experiment are shown in Table 4. The results show that all our proposed modules are useful and give performance gains to the local feature matching task. We can see that, without adding two modules, the precision is 85.05 and the AUC of $10^{\circ }$ is 44.16. When adding the AKGE module alone, the precision is 86.04 and the AUC of $10^{\circ }$ is 45.15. We can see that the AKGE module increases the precision by 0.99% and the AUC of $10^{\circ }$ by 0.99%. This result can show that dynamically adjusting the position of keypoints is crucial for the matching task. When adding the OGE module alone, the precision is 87.16 and the AUC of $10^{\circ }$ is 46.13. Compared to when no modules are added, the precision increases by 2.11%. The AUC value of $10^{\circ }$ is increased by 1.97%. It can be seen that the orientation of the keypoints can enhance the characterization of local features and have better performance in the case of large camera pose changes. When both modules are added simultaneously, the precision is 89.58 and the AUC for $10^{\circ }$ is 47.75. The overall precision is improved by 4.53% and the AUC value of $10^{\circ }$ is improved by 3.59%. These two modules have almost no burden on memory, which proves that our method works well in the task of local feature matching.

Not only do we need to know about the increase in accuracy, but we also need to understand the other conditions. In order to verify the time complexity and space complexity of our proposed modules, we compute the theoretical amount of multiply-add operations and the number of parameters. The experimental results are shown in Table 5.

## 6. Conclusions

We propose a method called geometric feature embedding matching (GFM). It can make full use of local features to model geometric information and to fuse local features and key geometric information, with the aim of enhancing representativeness and improving the accuracy and effectiveness of local feature matching. Our method achieves impressive results on all three large open datasets (MegaDepth, Hpatches, Aachen Day-Night v1.1), and is more competitive with most baseline methods. This result can prove the effectiveness of our proposed method. The ablation experiments we have done can well illustrate the effectiveness of the individual modules.

In the follow-up research, we aim to be able to add some parts of the downstream tasks (e.g., pose estimation) to the overall process to form an end-to-end approach.

## Figures and Tables

**Table 1:** Evaluation of State-of-the-art Approaches on MegaDepth.

Method	Pose est. AUC			Precision	MS
	@$5^{\circ }$	@$10^{\circ }$	@$20^{\circ }$		
R2D2 [18]	27.13	39.00	51.80	65.61	11.19
DISK [29]	28.68	40.90	52.04	64.61	21.22
SuperPoint [16]	22.08	33.93	45.97	44.01	15.33
DRC-Net [9]	20.91	32.32	45.36	-	-
SP [16] + SuperGlue [7]	36.40	46.10	56.85	87.16	25.40
Sparse-NCNet [22]	21.72	33.61	45.13	-	-
Ours	38.61	47.75	58.21	89.58	26.13

**Table 2:** Evaluation of State-of-the-art Approaches on HPatches.

Method	Homographt est. AUC		
	@3px	@5px	@10px
D2Net [17]	23.2	35.9	53.6
R2D2 [18]	50.6	63.9	76.8
DISK [29]	52.1	64.7	78.7
SuperPoint [16]	50.1	64.5	78.1
Sparse-NCNet [22]	48.9	54.2	67.1
DRC-Net [9]	50.6	56.2	68.3
Ours	51.9	65.8	79.1

**Table 3:** Evaluation of State-of-the-art Approaches on Aachen Day-Night v1.1.

Method	Day	Night
Loftr [27]	88.7/95.6/99.0	78.5/90.6/99.0
Rlocs [32]	88.8/95.4/99.0	74.3/90.6/98.4
R2D2 [18]	90.0/96.2/99.3	72.3/86.4/97.9
Patch2Pix [33]	86.4/93.0/97.5	72.3/88.5/97.9
SuperPoint [16] + SuperGlue [7]	89.8/96.1/99.4	77.0/90.6/99.5
Ours	90.2/96.4/99.5	74.0 / 91.5 / 99.5

**Table 4:** Effectiveness of Modules on MegaDepth.

AKGE	OGE	Pose est. AUC			Precision	MS
		@$5^{\circ }$	@$10^{\circ }$	@$20^{\circ }$		
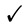	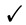	34.55	44.16	54.88	85.05	24.24
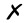	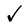	35.36	45.15	56.03	86.04	24.28
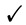	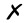	36.43	46.13	56.85	87.16	25.41
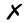	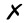	38.61	47.75	58.21	89.58	26.13

**Table 5:** Memory footprint and inference time of modules on MegaDepth.

Module	Precision	FLOPs (M)	Parameters (K)
AKGE	86.04	6.341	6.168
OGE	87.16	42.59	9445

## Data Availability

Not applicable.
